# A prognostic model integrating PET‐derived metrics and image texture analyses with clinical risk factors from GOYA

**DOI:** 10.1002/jha2.421

**Published:** 2022-03-24

**Authors:** Lale Kostakoglu, Federico Dalmasso, Paola Berchialla, Larry A. Pierce, Umberto Vitolo, Maurizio Martelli, Laurie H. Sehn, Marek Trněný, Tina G. Nielsen, Christopher R. Bolen, Deniz Sahin, Calvin Lee, Tarec Christoffer El‐Galaly, Federico Mattiello, Paul E. Kinahan, Stephane Chauvie

**Affiliations:** ^1^ Department of Radiology and Medical Imaging University of Virginia Charlottesville Virginia USA; ^2^ Medical Physics Division Santa Croce e Carle Hospital Cuneo Italy; ^3^ Department of Clinical and Biological Sciences University of Turin Turin Italy; ^4^ Department of Radiology University of Washington Seattle Washington USA; ^5^ Multidisciplinary Oncology Outpatient Clinic Candiolo Cancer Institute Candiolo Italy; ^6^ Hematology Department of Translational and Precision Medicine Sapienza University Rome Italy; ^7^ BC Cancer Center for Lymphoid Cancer and the University of British Columbia Vancouver British Columbia Canada; ^8^ 1st Faculty of Medicine Charles University General Hospital Prague Czech Republic; ^9^ F. Hoffmann‐La Roche Ltd Basel Switzerland; ^10^ Genentech, Inc. South San Francisco California USA; ^11^ Department of Hematology Aalborg University Hospital Aalborg Denmark

**Keywords:** diffuse large B‐cell lymphoma, imaging, lymphoid malignancies, quantitative PET, radiomics

## Abstract

Image texture analysis (radiomics) uses radiographic images to quantify characteristics that may identify tumour heterogeneity and associated patient outcomes. Using fluoro‐deoxy‐glucose positron emission tomography/computed tomography (FDG‐PET/CT)‐derived data, including quantitative metrics, image texture analysis and other clinical risk factors, we aimed to develop a prognostic model that predicts survival in patients with previously untreated diffuse large B‐cell lymphoma (DLBCL) from GOYA (NCT01287741). Image texture features and clinical risk factors were combined into a random forest model and compared with the international prognostic index (IPI) for DLBCL based on progression‐free survival (PFS) and overall survival (OS) predictions. Baseline FDG‐PET scans were available for 1263 patients, 832 patients of these were cell‐of‐origin (COO)‐evaluable. Patients were stratified by IPI or radiomics features plus clinical risk factors into low‐, intermediate‐ and high‐risk groups. The random forest model with COO subgroups identified a clearer high‐risk population (45% 2‐year PFS [95% confidence interval (CI) 40%–52%]; 65% 2‐year OS [95% CI 59%–71%]) than the IPI (58% 2‐year PFS [95% CI 50%–67%]; 69% 2‐year OS [95% CI 62%–77%]). This study confirms that standard clinical risk factors can be combined with PET‐derived image texture features to provide an improved prognostic model predicting survival in untreated DLBCL.

## 1 INTRODUCTION

Most patients with diffuse large B‐cell lymphoma (DLBCL) respond to current standard‐of care therapy with rituximab plus cyclophosphamide, doxorubicin, vincristine and prednisone (R‐CHOP)‐based regimens; however, approximately 20%–30% of patients relapse after initial response to first‐line therapy [1, 2]. The international prognostic index (IPI/R‐IPI) [3, 4, 5] and National Comprehensive Cancer Network‐IPI [6], which utilise only clinical factors, are routinely used to determine the prognosis of patients with DLBCL. However, advanced prediction models that include disease heterogeneity as a risk factor may provide enhanced patient risk stratification and improve patient outcomes.

The heterogeneous nature of DLBCL is reflected in transcriptionally defined tumour subtypes that can be classified based on cell‐of‐origin (COO). Previous studies have shown that molecular signatures can predict patient outcomes independent of the clinical IPI score and could help to identify patients suitable for targeted therapies [7, 8, 9]. The use of robust baseline biomarkers that predict response to immunochemotherapy is an important step towards improving baseline risk stratification and developing more efficacious treatment strategies for patients unlikely to benefit from R‐CHOP. Molecular imaging may also help separate patients into different prognostic groups that could lead to improved risk stratification.

Previous studies have demonstrated that baseline total metabolic tumour volume (TMTV) derived from fluoro‐deoxy‐glucose positron emission tomography/computed tomography (FDG‐PET/CT), is a useful prognostic indicator in DLBCL [10, 11, 12]. Although promising, the lack of methodological standardisation of TMTV measurements may limit its implementation as a prognostication tool in clinical practice [12].

While TMTV analysis enables determination of tumour burden, there is potential for extracting more substantial information from image data sets to improve evaluation of tumour heterogenicity and risk stratification in patients with DLBCL. Recently, radiographic image texture analysis, which can be defined as the interpretation of variations in image intensities, has become increasingly popular as a measure of tumour heterogeneity for predicting treatment response assessment and survival outcome. This strategy is in line with the hypothesis that the evaluation of tumour heterogeneity may be a noninvasive measure for determining biological characteristics of tumours [13]. Although image texture features analysis requires complex mathematical algorithms, this aspect should not constitute a barrier to accessing these techniques, as available advanced computer software programs can be employed to utilise these functions.

Image texture features can non‐invasively quantify tumour attributes, such as shape, intensity and heterogeneity that may be associated with clinical outcomes [14, 15]. While image texture features extracted using radiomics have the potential to be used as prognostic biomarkers, some methodological challenges remain, hindering the translation of this advanced methodology to clinical practice until a validated automated tool is developed [16]. Although radiomics have been studied in solid tumours [17], there are few studies investigating the use of radiomics in lymphoma [18, 19].

The objective of our study was to develop a prognostic model combining PET‐derived metrics, image texture features and clinical risk factors and determine whether this model could improve risk stratification compared with clinical factors alone, to predict progression‐free survival (PFS) and overall survival (OS) in patients with previously untreated DLBCL from the phase 3 GOYA trial (NCT01287741) [20].

## 2 METHODS

### 2.1 Patients

GOYA (NCT01287741) was an open‐label, multicentre, randomised, phase 3 trial designed to investigate the use of R‐CHOP or obinutuzumab plus cyclophosphamide, doxorubicin, vincristine and prednisone (G‐CHOP) in patients with previously untreated DLBCL. Details of the study design have been published in full previously [20]. In brief, patients were randomly assigned (1:1) to receive either eight 21‐day cycles of G or R, with six or eight cycles of CHOP. The GOYA trial (*N* = 1418) reported no significant treatment effect between the two arms [20], thus, the arms were combined for the present radiomics analysis. Data were included from patients with available baseline PET scans and detectable lesions within 1–35 days prior to randomisation.

GOYA was conducted in accordance with the Declaration of Helsinki and the International Conference on Harmonization guidelines for Good Clinical Practice.

### 2.2 COO analysis

COO classification (germinal centre B‐cell [GCB], activated B‐cell [ABC], or unclassified) was determined for biomarker‐evaluable patients (those with available tissue) using the research‐use‐only version of the NanoString Lymphoma Subtyping assay (NanoString Technologies, Inc., Seattle, WA, USA) [21, 22].

### 2.3 Clinical risk factors

The clinical parameters used as survival predictors included the IPI score, Ann Arbor stage, serum lactose dehydrogenase (LDH) level, extranodal involvement, Eastern Cooperative Oncology Group (ECOG) performance status, bulky disease status (lesion diameter ≥7.5 cm) and COO (Table 1). Ann Arbor stage was included both dichotomously and categorically. LDH level was included both dichotomously and continuously, for which the LDH level was divided by the upper limit of normal. Extranodal involvement and ECOG performance status were used with two different cut‐offs (extranodal involvement: ≥1 or >1 site, ECOG performance status: >1 or >2). For two patients whose performance status was missing, these values were imputed as ECOG performance status 0 for the IPI score. For the IPI prediction model, patients were divided into three prognostic IPI subgroups.

**TABLE 1:** Demographics and baseline characteristics of all patients and the COO subgroup

Characteristic n, (%)	All patients (*n* = 1263)	COO subgroup (*n* = 832)
Mean age (SD), y	59.4 (13.3)	60.6 (13.1)
Male	671 (53.1)	439 (52.8)
ECOG PS (>2)	161 (12.7)	105 (12.6)
Ann Arbor stage III/IV	1059 (83.8)	624 (75.0)
IPI score		
*Low‐intermediate*	701 (55.5)	448 (53.8)
*High‐intermediate*	367 (29.1)	252 (30.3)
*High*	195 (15.4)	132 (15.9)
Elevated serum LDH	728 (57.6)	493 (59.2)
Extranodal involvement (>1 site)	852 (67.4)	569 (68.4)
Bulky disease (≥7.5 cm)	462 (36.6)	322 (38.7)
COO		
*ABC*	–	213 (25.6)
*GCB*	–	481 (57.8)
*Unclassified*	–	138 (16.6)

Abbreviations: ABC, activated B‐cell like; COO, cell‐of‐origin; ECOG PS, Eastern Cooperative Oncology Group performance status; GCB, germinal centre B‐cell like; IPI, International Prognostic Index; LDH, lactate dehydrogenase; SD, standard deviation.

### 2.4 FDG‐PET/CT quality control and quantitative analysis

Baseline FDG‐PET/CT imaging was performed 1–35 days prior to randomisation (R‐CHOP or G‐CHOP) at study sites where a PET/CT scanner was available. All PET/CT scans were performed according to a standardised protocol, which has been described previously [20]. All imaging data were centrally collected with a quality control process in place, and only those scans with complete DICOM image sets were analysed. The quantitative PET analyses, including TMTV, were performed by an independent central review panel, and images were segmented using an adaptive method with a threshold equal to 1.5 times the mean standardized uptake value (SUV_mean_) of the liver, plus two standard deviations, using PET Encore workstation (MIM software Inc. Cleveland, Ohio). TMTVs were calculated as the sum of all the MTVs of the individual lesions.

### 2.5 Image feature extraction for radiomics analysis

Image texture features that represent tumour heterogeneity, as determined by mathematical modelling, were extracted from the FDG‐PET images. Using these features, tumour characteristics were analysed with the open‐source and validated PORTS radiomics toolkit as described in detail in the Supplemental Methods (Tables SI and SII). The radiomics analysis followed the framework of the Image Biomarkers Standardization Initiative [23].

### 2.6 Statistical analysis

Three prognostic models were used; two of these models were used to combine image texture features (radiomics), clinical risk variables and TMTV for survival analysis. The three prognostic models included: (1) IPI alone (2) random forest plot model [24] for survival analysis used on all variables and (3) Cox model applied to a subset of variables selected by random forest plot model. A bootstrap with a replacement validation approach was used [25]. The details of the Cox‐proportional model for survival analysis, and further rationale for the testing and validation approach, variable selection, patient risk stratification, and prognostic method comparison, are provided in the Supplemental Methods.

## 3 RESULTS

### 3.1 Patients

For the image texture analysis, 1263 patients had evaluable baseline PET scans, and COO data were available in a subgroup of 832 patients. Patient demographic and baseline characteristics were similar between the total GOYA patient population (data not shown) [20], the subgroup population in the present analysis and the COO subgroup (Table 1). The median PFS follow‐up time was 44.5 months (range, 1–74 months), and the median OS follow‐up time was 48.2 months (range, 1–74 months) for all patients. The median TMTV was 357 cm^3^ for all patients and 350 cm^3^ for the COO subgroup.

### 3.2 Selection of the prognostic variables and validation of the prognostic model

The random forest plot method identified the 10 most significant variables associated with patient survival (Table SI). Association with PFS and OS was subsequently confirmed using a multivariate Cox regression analysis (Supplemental Section). Hazard ratios (HRs) from the multivariate analysis of risk factors for PFS and OS in the total population and COO subgroup are shown in Tables SI and SII. The nomograms obtained using Cox regression analysis for PFS in the total population and COO subgroup are shown in Figure S2.

In the entire patient population, for the constructed prognostic model, the bootstrapped Brier score and c‐index were 0.32 and 0.60 for PFS, and 0.44 and 0.62 for OS, respectively. In the COO subgroup, the bootstrapped Brier score and c‐index were 0.39 and 0.64 for PFS and 0.22 and 0.66 for OS, respectively.

All the clinical and radiomics variables and TMTV were inserted into a random forest model for survival, regression and classification. In the entire cohort, the percentage error in describing PFS and OS was 44.0% and 45.2%, respectively; in the COO subgroup, the percentage error in describing PFS and OS was 42.9% and 42.8%, respectively.

### 3.3 Identification of risk groups and probability of survival

The probability of survival at 2 years for the entire cohort and the COO subgroup was determined based on the three IPI risk groups (high, high‐intermediate and low/low‐intermediate) alone and also using a random forest model combining IPI, TMTV, all image texture features and COO, by dividing the patients into three prognostic subgroups for treatment‐failure risk: low, intermediate and high (Table 2). Notably, adding COO to the created model trumped the prognostic value of IPI, with respect to the prediction of PFS (Table SI).

**TABLE 2:** Survival probabilities at 2 years for IPI and random forest model. Stratification into risk groups was carried out separately for all patients and for the subgroup of patients with COO data

	IPI, % (95% CI)	Random forest, % (95% CI)
**All patients (*N* = 1263)**		
PFS		
*Low risk*	79 (76–82)	94 (91–96)
*Intermediate risk*	70 (65–75)	72 (67–76)
*High risk*	59 (52–67)	54 (50–60)
OS		
*Low risk*	89 (86–91)	100 (100–100)
*Intermediate risk*	82 (78–86)	100 (100–100)
*High risk*	72 (65–78)	51 (46–56)
**COO subgroup analysis (*n* = 832)**		
PFS		
*Low risk*	80 (77–84)	88 (84–92)
*Intermediate risk*	70 (64–76)	86 (82–91)
*High risk*	58 (50–67)	45 (40–52)
OS		
*Low risk*	88 (85–92)	91 (88–95)
*Intermediate risk*	81 (76–86)	93 (90–96)
*High risk*	69 (62–77)	65 (59–71)

Abbreviations: CI, confidence interval; COO, cell‐of‐origin; IPI, International Prognostic Index; OS, overall survival; PFS, progression‐free survival.

**FIGURE 1:**
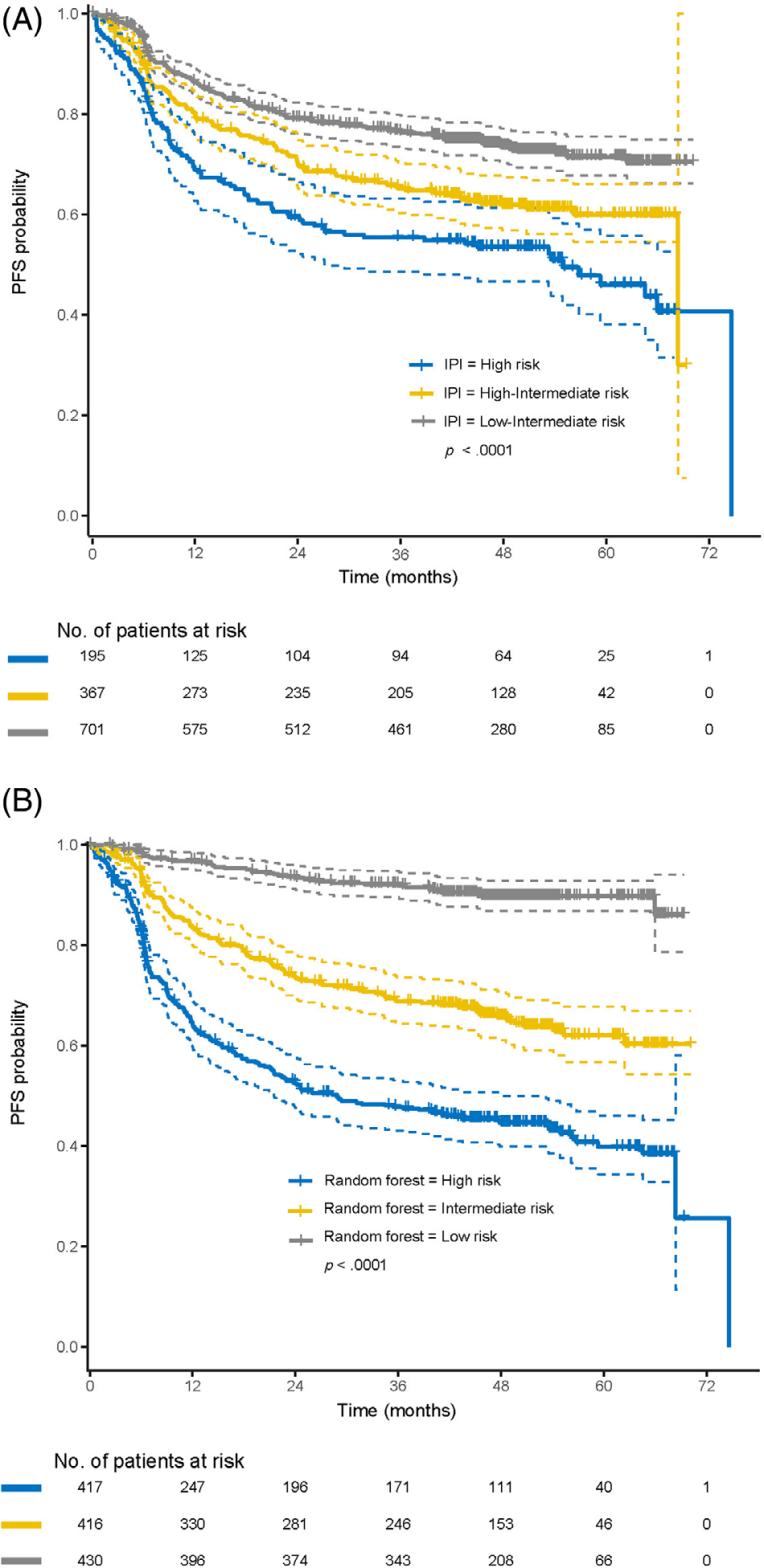
Kaplan–Meier PFS curves for the three risk groups as defined by (A) IPI and (B) random forest prediction model in all patients. IPI, international prognostic index; PFS, progression‐free survival. Note: Dashed lines indicate the 95% confidence intervals

**FIGURE 2:**
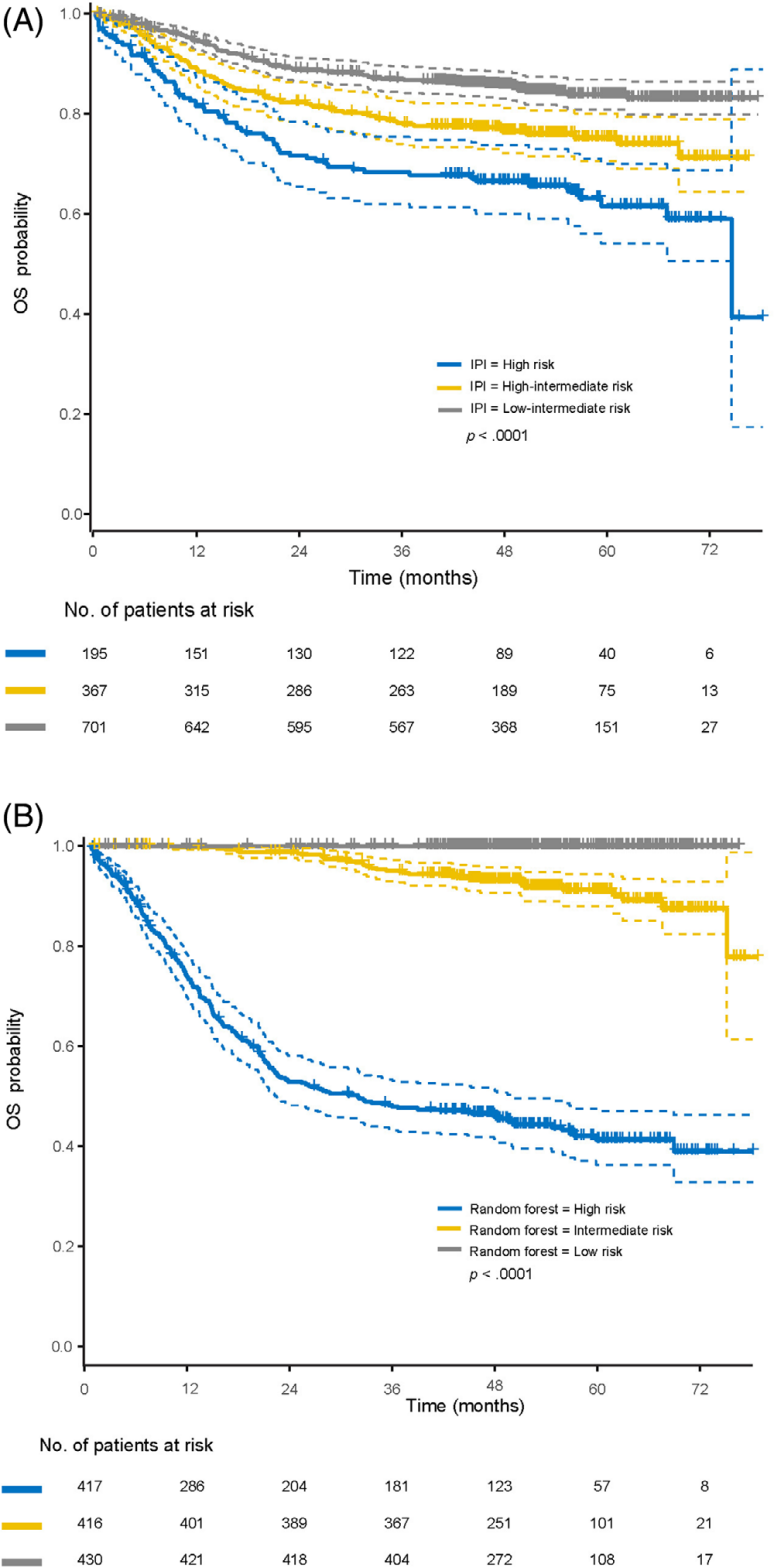
Kaplan–Meier OS curves for the three risk groups as defined by (A) IPI and (B) random forest prediction model in all patients. IPI, international prognostic index; OS, overall survival. Note: Dashed lines indicate the 95% confidence intervals

**FIGURE 3:**
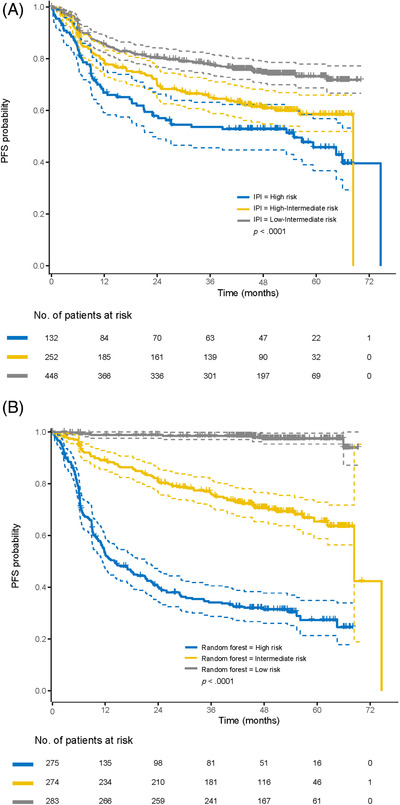
Kaplan–Meier PFS curves for the three risk groups as defined by (A) IPI and (B) random forest prediction model for the COO subgroup. COO, cell‐of‐origin; IPI, international prognostic index; PFS, progression‐free survival. Note: Dashed lines indicate the 95% confidence intervals

**FIGURE 4:**
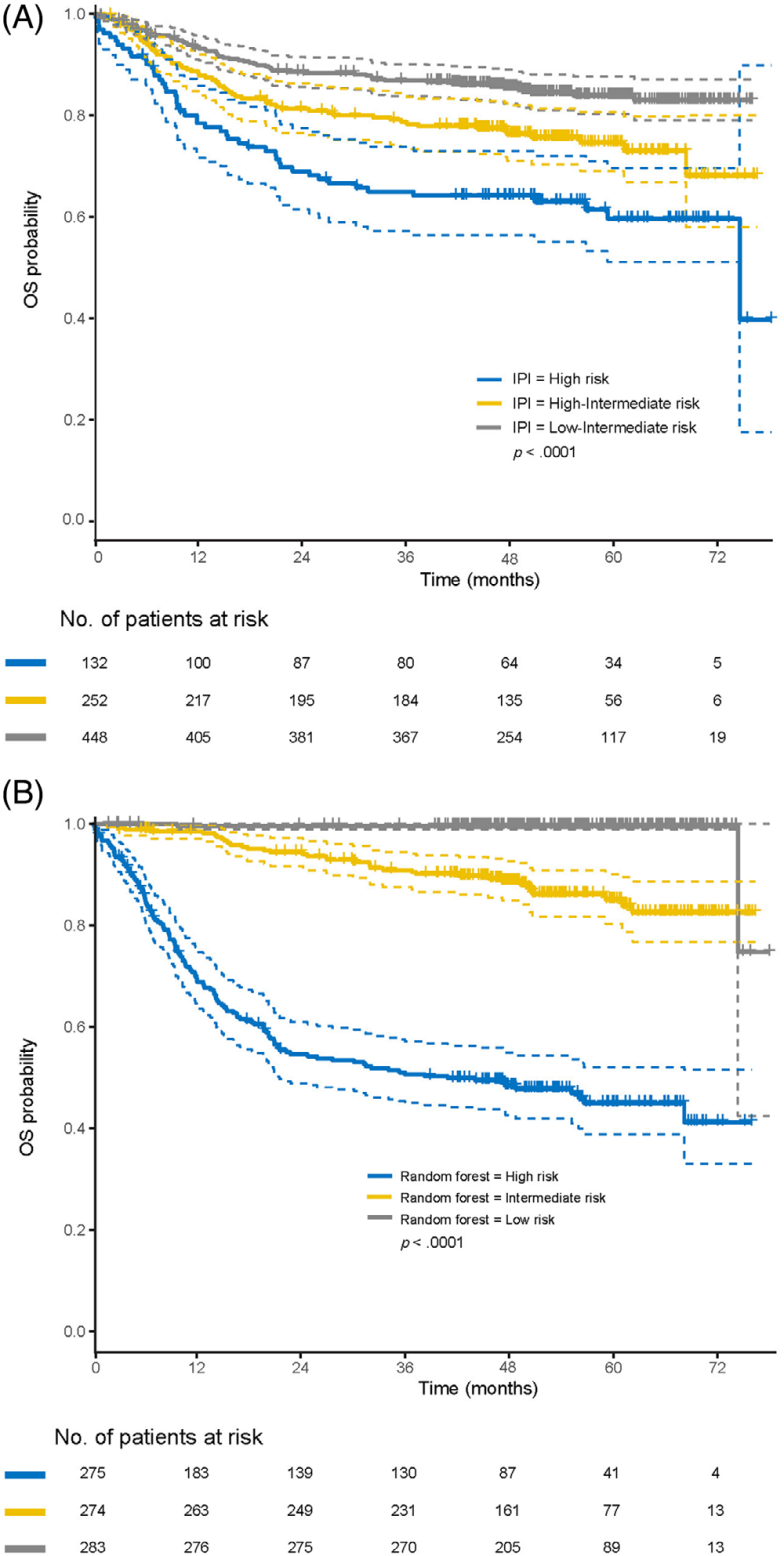
Kaplan–Meier OS curves for the three risk groups as defined by (A) IPI and (B) random forest prediction model for the COO subgroup. COO, cell‐of‐origin; IPI, International Prognostic Index; OS, overall survival. Note: Dashed lines indicate the 95% confidence intervals

The 2‐year survival probability of the three predictive models (as described in the methods section), stratified by IPI risk groups is shown in Table SIII. Generally, probabilities predicted by the Cox regression analysis with variable selection were similar to those predicted by the IPI. However, survival probability in the low‐risk group as predicted by the random forest model was consistently higher than that by other models (e.g., 2‐year PFS of 94% [95% confidence interval [CI] 91–96] vs. 79% [95% CI 76–82] for the IPI and 80% [95% CI 77–84] for the Cox analysis). Additionally, the random forest model with COO subgroups identified a more clearly defined high‐risk population (45% 2‐year PFS [95% CI 40%–52%]; 65% 2‐year OS [95% CI 59%–71%]) than the IPI (58% 2‐year PFS [95% CI 50%–67%]; 69% 2‐year OS [95% CI 62%–77%]). Kaplan–Meier curves for PFS and OS are shown in Figures 1 and 2 for all patients stratified by low, intermediate and high risk of treatment failure, predicted by IPI risk classification and random forest model for all variables. Predictions for the COO subgroup are shown in Figures 3 and 4. Kaplan–Meier curves for PFS and OS for the corresponding Cox regression analyses are shown in the Supplemental Section (Figures S3–S6).

### 3.4 Comparison of different prognostic models

The receiver operator characteristics (ROCs) analysis was applied to the three distinct prognostic models. The calculated area under the curve (AUCs) for PFS and OS are shown in Table 3. The results of the AUC analysis were superior for the random forest model compared with the Cox regression analysis and IPI risk classification. Notably, a model based on TMTV alone demonstrated comparable results to the Cox model and IPI model but was inferior to the random forest model (data not shown).

**TABLE 3:** AUC from ROC analysis using IPI, Cox, and random forest for PFS and OS

	IPI, % (95% CI)	Cox, % (95% CI)	Random forest, % (95% CI)
**All patients (*N* = 1263)**			
** PFS**	0.55 (0.53–0.58)	0.63 (0.60–0.66)	0.74 (0.76–0.99)
** OS**	0.57 (0.54–0.60)	0.63 (0.59–0.66)	0.92 (0.91–0.94)
**COO subgroup analysis (*n* = 832)**			
** PFS**	0.57 (0.55–0.60)	0.66 (0.61–0.69)	0.86 (0.83–0.88)
** OS**	0.58 (0.54–0.61)	0.71 (0.67–0.75)	0.89 (0.87–0.91)

Abbreviations: AUC, area under the curve; CI, confidence interval; COO, cell of origin; IPI, international prognostic index; OS, overall survival; PFS, progression‐free survival; ROC, receiver operator characteristics.

## 4 DISCUSSION

In this study, we developed a prognostic model for PFS and OS, combining intratumour heterogeneity features and PET‐derived metrics, alongside other clinical risk factors, in previously untreated patients with DLBCL. Image texture features may reflect tumour heterogeneity, allowing for identification of tumour subregions with different phenotypes that may be associated with varying treatment outcomes. Hence, this advanced methodology potentially offers a powerful tool for the extraction of clinically relevant prognostic information, otherwise not readily detectable for DLBCL, which is renowned for its phenotypical heterogeneity. We found that radiomic features, more specifically image texture features, extracted from FDG‐PET data, predicted PFS, and in combination with other known risk factors, improved the predictive value for PFS, when compared with traditional clinical risk factors alone, in previously untreated patients with DLBCL who received immunochemotherapy. However, individual image features remain elusive as they do not directly correlate with biological features that can be translated to a clinical decision‐making process. To our knowledge, this is the first study to analyse radiomics data from a large cohort of patients with DLBCL participating in a prospective study.

Regarding other PET‐based quantitative parameters, previous studies have investigated the utility of TMTV as a predictor of survival in patients with DLBCL treated with R‐CHOP [26]. While data are mainly retrospective and utilised variable segmentation thresholds, one of the radiomic features, TMTV, has been consistently reported to have prognostic value; patients with a high TMTV are expected to have lower PFS and OS compared with those with a low TMTV. The present analysis indicated a similar effect of TMTV. A review of seven retrospective studies in patients with DLBCL (*n* = 703) [27] also revealed a significant prognostic value of TMTV for PFS (HR = 2.18; *p* = 0.000); 3‐year OS was unfavourably impacted by high TMTV (odds ratio, 5.40). Different risk scoring systems impacted the homogeneity of the analysis; moreover, each study varied widely in the optimal cut‐off values for survival prediction, with cut‐off values ranging from 11 to 30 for ΔSUV_max_, and 220 ml to 550 ml for MTV. The small sample size may have influenced the reliability of these results.

In a previous analysis of the GOYA trial, baseline TMTV was shown to be an independent predictor of PFS (HR = 2.21, *p* < 0.0001); however, SUV_max_ was not a reliable predictor of PFS (*p* = 0.38) [28]. Our findings are consistent with this previous analysis, having demonstrated that standard PET‐derived metrics are prognostic for survival. The previous analysis of the GOYA trial also investigated patients with available COO data (*n* = 880) [28]. TMTV was identified as being more prognostic in patients with ABC and unclassified DLBCL subtypes (HR = 3.08, *p* = 0.0012) versus those with GCB DLBCL (HR = 2.30, *p* = 0.0176). This suggests that within this population, there is differentiation in outcomes, even in the higher‐risk group, that can be identified through prognostic markers such as TMTV. Interestingly, in our study, adding COO to this model overcame the prognostic value of IPI with respect to prediction of PFS. Such insights could be used as a factor for improved patient management algorithms.

In this study, we prioritised the investigation of the predictive value of image texture features as a measure of tumour heterogeneity. Previous studies have indicated an association between radiomics features and genetics in lymphoma, and it has been hypothesized that tumour heterogeneity, as described at the cellular level, can be partly captured through radiomics analysis, particularly, PET‐based image textural analysis [29, 30, 31]. One of the first works investigating the prognostic value of radiomics was conducted in a small, mixed population of 57 patients with Hodgkin and Non‐Hodgkin lymphoma [32]. The study found that the addition of radiomics features to TMTV and histology increased AUC in the early response evaluation. In another study of patients with primary mediastinal B‐cell lymphoma (*n* = 103) enrolled in a prospective multicentre clinical trial (IELSG26), AUC cumulative SUV‐volume histograms discriminated between two groups of patients with different prognoses [33]. In the present study, the use of several image texture features was found to be prognostic for PFS and OS in patients with DLBCL.

While studies in patients with DLBCL are scarce and often retrospective, baseline FDG‐PET heterogeneity evaluated by radiomics features has been found to be a promising predictor of objective response at end‐of‐treatment with PET evaluated using the Lugano classification [34], to provide a detailed assessment of bone marrow involvement [35], and to increase the predictive power of TMTV [36]. More recently, a retrospective cohort of 132 patients with DLBCL found that multivariable analysis, including IPI and TMTV, image texture features (long‐zone high‐grey level emphasis) was the only independent predictor of 2‐year event‐free survival [19]. Similarly, a radiomics‐based model integrating baseline FDG‐PET radiomics signatures and clinical factors yielded good predictive values for survival of 110 patients with nasal‐type extranodal natural killer/T‐cell lymphoma [37]. Nonetheless, superiority for the radiomics model could not be demonstrated when compared with traditional semi‐quantitative imaging features including TMTV and SUVs.

The quality of published radiomics studies in lymphoma has been variable, which was also confirmed in a recent systematic review [38]. Most studies to date have had insufficient samples for analysis [35, 36], patients undergoing mixed treatments, limited statistical analysis [35] and other methodological shortcomings [36] that limit the applicability of results. In a study by Cotterau *et al* [18]. Four dissemination features were investigated in 95 patients enrolled in the LNH073B trial with DLBCL, two groups with better PFS and OS separation with respect to TMTV were discerned using one of the radiomics features, D_max_ (the maximum distance among lesions). Combining TMTV and D_max_ enabled patients with a poor prognosis to be identified by physicians so that they may consider changes to their treatment. Indeed, patients with high baseline MTV (>394 cm^3^) and high D_max_ (>58 cm) had a poor prognosis. In the present study, survival predicted using the Cox regression analysis generally appeared similar to that predicted by IPI scores. However, survival rates in the high‐risk group predicted by random forest model were consistently lower than those predicted by IPI. Survival probabilities generated using IPI appear more similar to those predicted using the Cox regression analysis compared with the random forest model.

In summary, in the present study we generated a prognostic model for PFS and OS in previously untreated patients with DLBCL using intratumour heterogeneity features derived from radiomics analyses of PET scans, as well as PET‐derived metrics and other clinical risk factors. For both the full cohort and COO subgroups, the Cox model using random survival forest models for variable selection was significantly more prognostic than IPI for PFS and OS. PET‐derived image texture features, in combination with more common clinical risk factors, were able to predict survival probability for untreated DLBCL patients with good precision. The results of this study strongly suggest that a PET‐based prognostic model, with further validation, may help to identify patients at diagnosis who are at greater risk of treatment failures with standard therapy (R‐CHOP). The individual treatment strategy for these patients could utilise this prognostic model for personalised, novel treatment approaches. A variety of challenges remain to be addressed in the field of radiomics, including facilitation and standardisation of all stages of the workflow, development of a more comprehensible algorithm to provide an improved clinical model, and in common with all quantitative metrics, labour intensity. In the future, it would seem likely that artificial intelligence and machine‐learning methods will play a larger part in strengthening radiomics research and accelerating clinical translation, providing more robust and practical workflows that support the use of radiomics as a clinical endpoint.

## CONFLICT OF INTEREST

LK is a consultant at F. Hoffmann‐La Roche Ltd, Genentech, Inc., and reports travel, accommodations and other expenses to F. Hoffmann‐La Roche Ltd. LAP reports equity ownership in Precision Sensing LLC. UV reports a consulting or advisory role for Janssen, Celgene, Juno Therapeutics and Kite Pharma; speaker's bureau fees from F. Hoffmann‐La Roche Ltd, Janssen, Celgene, Gilead Sciences, Servier and AbbVie; research funding from Celgene; and travel, accommodations or other expenses from Celgene, F. Hoffmann‐La Roche Ltd and AbbVie. MM has served on a consulting and advisory board and speaker's bureau for F. Hoffmann‐La Roche Ltd, Janssen, Novartis, Gilead Sciences and Sandoz; and reports travel, accommodations and other expenses from F. Hoffmann‐La Roche Ltd. LHS reports research funding from F. Hoffmann‐La Roche Ltd and Genentech, Inc. and consulting and honoraria fees from F. Hoffmann‐La Roche Ltd, Genentech, Inc., AbbVie, Amgen, Apobiologix, Acerta, AstraZeneca, Celgene, Gilead Sciences, Janssen, Kite Pharma, Karyopharm, Lundbeck, Merck, MorphoSys, Seattle Genetics, Takeda, Teva, TG Therapeutics and Verastem. MT reports honoraria and consulting fees from Janssen, Gilead Sciences, Bristol‐Meyers Squibb, Amgen, AbbVie, Takeda, F. Hoffmann‐La Roche Ltd, MorphoSys and Incyte; consulting for Celgene; and travel, accommodation and other expenses from AbbVie, Gilead Sciences, Bristol‐Meyers Squibb, Takeda, F. Hoffmann‐La Roche Ltd and Janssen. TGN is an employee and stockholder of F. Hoffmann‐La Roche Ltd. CRB is an employee of Genentech, Inc. and stockholder of F. Hoffmann‐La Roche Ltd. DS is an employee and stockholder of F. Hoffmann‐La Roche Ltd. CL is an employee of Genentech, Inc. TCE‐G is a former employee of F. Hoffmann‐La Roche Ltd and reports speaker fees for AbbVie. FM is an employee of F. Hoffmann‐La Roche Ltd. SC reports research funding from F. Hoffmann‐La Roche Ltd. and honoraria fees from Sirtex Medical. FD, PB and PEK have declared no conflict of interest.

## AUTHOR CONTRIBUTIONS

LK and SC designed the study. LK, FD, PEK and SC, conducted the study. UV, MM, LHS and MT were responsible for the recruitment and follow‐up of patients. LK, FD and SC collected the data. LK, FD, PB, LAP, CRB and SC analysed the data. TGN, DS, CRB, CL, TCE‐G and FM interpreted the data. All authors critically reviewed and edited the manuscript, provided their final approval of the manuscript and are accountable for all aspects of the work.

## ETHICS STATEMENT

The protocol was approved by the ethics committees at participating centres. All patients provided written informed consent.

## Supporting information

SUPPORTING INFORMATION

## Data Availability

For eligible studies, qualified researchers may request access to individual patient level clinical data through a data request platform. At the time of writing this paper, request platform is Vivli: https://vivli.org/ourmember/roche/. For up‐to‐date details on Roche’s Global Policy on the Sharing of Clinical Information and how to request access to related clinical study documents, see: https://go.roche.com/data_sharing
