# Supplementary material for: A prognostic model integrating PET‐derived metrics and image texture analyses with clinical risk factors from GOYA
Source: EJHaem. 2022 Mar 24;3(2):406–14. doi: 10.1002/jha2.421 (PMC9175666; doi:10.1002/jha2.421)
Supplement: Supplementary file 1 — SUPPORTING INFORMATION [file JHA2-3-406-s001.docx]

**SUPPLEMENTAL MATERIAL**

**METHODS***Image texture features*

Image texture features were extracted from the fluoro-deoxy-glucose positron emission tomography (FDG-PET) images to analyse tumour characteristics. They were calculated using the open-source and validated PET Oncology Radiomics Test Suite (PORTS) toolkit.^1,2^ The original pixel size was converted to a standard isotropic voxel of 4×4×4 mm^3^ through bicubic spline interpolation. Image pixel values were discretised by a fixed number of 64 bins between maximum and minimum to avoid dependence of radiomics texture from pixel values itself. Forty-two features^1^ were extracted from the images, including histogram features and four textural matrices: the grey-level co-occurrence matrix; neighboring grey-level dependence matrix; grey-level run-length matrix; and grey-level size zone matrix. The radiomics metrics were calculated on the tumour mass with the largest size and highest maximum standard unit value (SUV_max_) and with similar size lesions; only those with the highest uptake were analysed. For radiomics analysis a minimal volume of 1 ml was required.

*Rationale for the testing and validation approach*

Model performance can be approached in different ways. External validity can be assessed using independent cohorts that the model has not previously seen during the training phase. One example of this is the study by Aerts *et al.* that evaluated the prognostic ability of a predictive model based on computed tomography-derived radiomics features for patients with lung or head and neck cancer.^3^ In the study by Aerts *et al.*, the model was trained using a cohort of *N* = 422 patients and then evaluated using four additional cohorts with a total of *N* = 545 patients. However, for the GOYA study using PET imaging, no equivalent external cohorts were available for validation.

Another approach for validation is the split-sample approach, for example training on arm one of the GOYA study and validation on arm two. However, it has been shown that a split-sample approach with 50% held out leads to models with the same performance as obtained with half the sample size, and a bootstrap with replacement validation approach is recommended.^4-6^

*Cox-proportional model for survival analysis*

The prognostic model was obtained in two ways as depicted in Supplemental Fig S1. In the statistical analysis section below, we present risk-group prediction carried out using the random forest model. Here, we present extensive data from the Cox regression analysis.

*Methodology.*

Due to the large number of variables (radiomics features) relative to the sample size, the capability to uncover potential non-linear signals in the data and potential interactions, Random Survival Forest-Variable Hunting (RFS-VH) regularization algorithm was preferred to least absolute shrinkage and selection operator for identifying the most prognostic factors for progression-free survival (PFS) and overall survival (OS). The variables selected by RFS-VH were then entered into a prognostic multivariate model (one for PFS and one for OS) based on Cox regression analysis. The non-linear effect of covariates was modelled using restrictive cubic spline functions, and its significance was assessed by Wald test. Interaction among variables was checked similarly using the Wald test. Proportional hazards assumptions were checked by inspection of the scaled Schoenfeld residuals. Furthermore, the statistical model predictive accuracy evaluation was based on c-index (the closer to 1, the better) and Brier score (the closer to 0, the better). The hazard ratio (HR) and associated 95% confidence interval (CI) together with the *p*-value of the log-rank test were also reported. In the absence of an independent patient cohort for external model validation, to account for the degree of optimism in model accuracy due to the use of the same data for training and testing purpose, c-index and Brier score indexes were computed using bootstrap (5000 runs). To check the potential bias of estimates of risk, a bootstrap optimism corrected calibration of risk prediction for a single time point was assessed by resampling the differences between average Cox predicted survival and Kaplan-Meier estimates.

In the nomogram, a risk score for each patient was calculated based on the linear predictor from the results of Cox regression analysis fitting functions. Since the proportion of patients with no event in respect to those with event is about 2/3, we created three risk populations grouping patients with a predicted probability of event lower than 33% (low risk), those with a probability between 33% and 66% (intermediate risk) and those with a probability of an event higher than 66% (high risk). This was also done to mimic and compare with the predictions obtained for the three International Prognostic Index (IPI) groups.

The most important variables identified by the random forest model were used to construct the Cox proportional model. HRs for PFS are shown in Supplemental Table SI and HRs for OS are shown in Supplemental Table SII. In the entire population, bootstrapped Brier score and c-index were 0.32 and 0.60 for PFS, and 0.44 and 0.62 for OS, respectively. In the cell-of-origin (COO) subgroup, Brier score and c-index were 0.39 and 0.64 for PFS, and 0.22 and 0.66 for OS, respectively. The graphical representation of the nomograms is shown in Supplemental Fig S2.

Based on IPI, total metabolic tumour volume radiomics features and genetic characteristics, in the COO population, patients were divided into three prognostic subgroups: low-, intermediate- and high-risk as in the main paper. The groups were predicted using the nomogram obtained with the Cox regression analysis. The Kaplan-Meier survival curves for PFS and OS are shown in Supplemental Figs S3 and S4 for all patients, and in Supplemental Figs S5 and S6 for the COO subgroup.

*Statistical analysis*

*Variable selection*.

Due to a large number of image texture features relative to the sample size, the capability to uncover potential non-linear signals and interactions in the data was constrained. Therefore, to identify the most significant prognostic factors for PFS and OS, the Random Survival Forest-Variable Hunting regularization algorithm^6^ was chosen over least absolute shrinkage and selection operator.

*Risk stratification of patients.*

A model was created to determine patient outcome stratification using the random forest model. The random forest model creates survival probability for each patient for each time point based on all parameters inserted in the model as continuous parameters without classifying into groups.^7^ Consequently no survival curves are created based on a predetermined cut-off for any parameter. In this data set, for the purpose of demonstrating survival differences using the risk models such as IPI, the patient population was divided into three risk groups predicted by the random forest model, as high-, intermediate- and low-risk levels; low risk: with a predicted probability of an event lower than 33%; intermediate risk: those with a predicted probability between 33% and 66%; and high risk: those with a probability of an event greater than 66%.

*Comparison of the prognostic methods.*

The area under the curve of receiver operator characteristics was computed to compare the performance of the IPI, random forest (for all variables) and Cox (for selected variables) prognostic models. The CIs were calculated on 2000 stratified bootstrap replicates.

**References**

1. Hatt M, Tixier F, Pierce L, Kinahan PE, Le Rest CC, Visvikis D. Characterization of PET/CT images using texture analysis: the past, the present… any future? *Eur J Nucl Med Mol Imaging*. 2017;**44**(1):151-65.

2. Wu W, Pierce LA, Zhang Y, Pipavath SNJ, Randolph TW, Lastwika KJ, et al. Comparison of prediction models with radiological semantic features and radiomics in lung cancer diagnosis of the pulmonary nodules: a case-control study. *Eur Radiol*. 2019;**29**(11):6100-8.

3. Aerts HJWL, Velazquez ER, Leijenaar RTH, Parmar C, Grossmann P, Carvalho S, et al. Decoding tumour phenotype by noninvasive imaging using a quantitative radiomics approach. *Nature Communications*. 2014;**5**(1):4006.

4. Steyerberg EW. Validation in prediction research: the waste by data splitting. *J Clin Epidemiol*. 2018;**103**:131-3.

5. Austin PC, Steyerberg EW. Events per variable (EPV) and the relative performance of different strategies for estimating the out-of-sample validity of logistic regression models. *Stat Methods Med Res*. 2017;**26**(2):796-808.

6. Ishwaran H, Kogalur UB, Gorodeski EZ, Minn AJ, Lauer MS. High-Dimensional Variable Selection for Survival Data. *J Am Stat Assoc*. 2010;**105**(489):205-17.

7. Ishwaran H, Kogalur U, Blackstone E, Lauer M. Random survival forests. *Ann Appl Stat*. 2008;**2**(3):841-60.

**Table SI.** Multivariate analysis of risk factors for PFS using Cox regression analysis. *p*‑values for significant risk factors are indicated in bold text. Some variables (designated NA) were not selected by the random forest model).

|  | All patients  (*n* = 1250) | | Patients with COO  (*n* = 832) | | |
| --- | --- | --- | --- | --- | --- |
|  | **HR (95% CI)** | ***p*-value** | **HR (95% CI)** | ***p-value*** |  |
| IPI |  |  |  |  |  |
| *High-intermediate vs. low-intermediate* | 1.40 (1.10–1.79) | **0.007** | NA |  |  |
| *High vs. low-intermediate* | 1.81 (1.37–2.40) | **< 0.001** | NA |  |  |
| COO |  |  |  |  |  |
| *ABC vs. GCB* | NA |  | 1.58 (1.14–2.20) | **0.006** |  |
| *Unclassified vs. GCB* | NA |  | 1.31 (0.88–1.95) | 0.178 |  |
| BCL2 (positive vs. negative) | NA |  | 1.47 (1.08–1.98) | **0.013** |  |
| TMTV (high vs. low) | 1.14 (1.04–1.24) | **0.003** | 1.17 (1.07–1.29) | **< 0.001** |  |
| Histogram mean | NA |  | 0.78 (0.57–1.06) | 0.114 |  |
| ITF (high vs. low) |  |  |  |  |  |
| GTSDM  *Sum average* | 1.44 (1.01–2.04) | **0.042** | NA |  |  |
| *Difference variance* | 1.07 (0.81–1.41) | 0.639 | NA |  |  |
| *Correlation* | NA |  | 1.44 (0.93–2.23) | 0.105 |  |
| *Inverse difference* | 1.24 (1.03–1.50) | **0.023** | NA |  |  |
| NGTDM  *Texture strength* | NA |  | 0.87 (0.77–1.00) | **0.041** |  |
| *Busyness* | 1.01 (1.00–1.03) | **0.068** | 1.01 (1.00–1.03) | 0.140 |  |
| GLSZM  *Large zone low grey emphasis* | 1.00 (1.00–1.00) | 0.419 | 1.00 (1.00–1.00) | 0.435 |  |
| *Zone size percentage* | NA |  | 1.15 (0.69–1.92) | 0.591 |  |
| *Zone size non uniformity* | 0.93 (0.82–1.05) | 0.260 | NA |  |  |
| *Large zone high grey emphasis* | 1.00 (1.00–1.00) | 0.126 | 1.00 (0.99–1.00) | 0.241 |  |

ABC, activated B-cell like; BCL2, B-cell lymphoma 2; CI, confidence interval; COO, cell-of-origin; GCB, germinal centre B-cell like; GLSZM, low grey-level zone length matrix; GTSDM, grey-tone spatial dependence matrices; HR, hazard ratio; IPI, International Prognostic Index; ITF, image texture features; NA, not applicable; NGTDM, neighboring grey-tone difference matrix; PFS, progression-free survival; TMTV, total metabolic tumour volume.

**Table SII**. Multivariate analysis of risk factors for OS using Cox regression analysis. *p*‑values for significant risk factors are indicated in bold text. Some variables (designated NA) were not selected by the random forest model).

|  | All patients (*n* = 1250) | | Patients with COO (*n* = 832) | |
| --- | --- | --- | --- | --- |
|  | **HR (95% CI)** | ***p*-value** | **HR (95% CI)** | ***p-value*** |
| IPI |  |  |  |  |
| *High-intermediate vs. low-intermediate* | 1.40 (1.03–1.90) | **0.032** | 1.20 (0.69–2.09) | 0.524 |
| *High vs. low-intermediate* | 2.02 (1.44–2.83) | **< 0.001** | 1.67 (0.87–3.21) | 0.121 |
| COO |  |  |  |  |
| *ABC vs. GCB* | NA |  | 1.88 (1.10–3.26) | **0.021** |
| *Unclassified vs. GCB* | NA |  | 1.69 (0.89– 3.21) | 0.110 |
| Double-hit lymphoma  (positive vs. negative) | NA |  | 5.65 (2.39–13.38) | **< 0.001** |
| TMTV (high vs. low) | 1.18 (1.09–1.28) | **< 0.001** | 1.91 (1.40–2.62) | **< 0.001** |
| ITF (high vs. low)  GTSDM  *Cluster shade* | 1.10 (0.93–1.29) | 0.264 | 1.52 (1.07–2.15) | **0.018** |
| *Angular second moment* | 0.99 (0.98–1.01) | 0.415 | 1.52 (1.07– 2.15) | **0.018** |
| *Inverse difference* | 1.10 (0.95–1.27) | 0.215 | NA |  |
| *Information correlation 2* | NA |  | 0.62 (0.35–1.08) | 0.089 |
| *Sum variance* | NA |  | 1.40 (0.99–1.97) | 0.056 |
| NGTDM  *Texture strength* | 0.87 (0.77–1.00) | 0.045 | NA |  |
| *Busyness* | 1.01 (0.99–1.03) | 0.225 | 1.02 (0.99–1.04) | 0.148 |
| *Coarseness* | 1.06 (0.98–1.15) | 0.128 | NA |  |
| GLSZM  *Large zone low grey emphasis* | 1.00 (1.00–1.00) | **0.003** | 1.00 (1.00–1.00) | **0.014** |
| *Large zone high grey emphasis* | 0.99 (0.99–1.00) | **0.002** | 0.99 (0.97–1.01) | 0.453 |
| *Grey level non uniformity* | NA |  | 0.59 (0.42–0.84) | **0.003** |
| ITF GLSZM zone size non uniformity (high vs low) | 1.12 (0.88; 1.43) | 0.35 |  |  |

ABC, activated B-cell like; CI, confidence interval; COO, cell-of-origin; GCB, germinal centre B-cell like; GLSZM, low grey-level zone length matrix; GTSDM, grey-tone spatial dependence matrices; HR, hazard ratio; IPI, International Prognostic Index; ITF, image texture features; NA, not applicable; NGTDM, neighboring grey-tone difference matrix; OS, overall survival; TMTV, total metabolic tumour volume.

**Table SIII.** Survival probabilities at 2 years for IPI, Cox regression analysis and random forest model. IPI and random forest model are the same as in the main manuscript and are provided for comparison. Stratification in risk groups has been carried out separately for the all patients model and for the COO subgroup.

|  | IPI, %  (95% CI) | Cox, %  (95% CI) | Random forest, % (95% CI) |  |
| --- | --- | --- | --- | --- |
| All patients (*N* = 1250) |  | | | |
| PFS |  | | | |
| *Low risk* | 79 (76–82) | 80 (77–84) | 94 (91–96) |  |
| *Intermediate risk* | 70 (65–75) | 78 (74–82) | 72 (67–76) |  |
| *High risk* | 59 (52-67) | 61 (57–66) | 54 (50–60) |  |
| OS |  | | | |
| *Low risk* | 89 (86–91) | 89 (86–92) | 100 (100–100) |  |
| *Intermediate risk* | 82 (78–86) | 90 (87–93) | 100 (100–100) |  |
| *High risk* | 72 (65–78) | 74 (70–78) | 51 (46–56) |  |
| COO subgroup  (*n* = 832) |  | | | |
| PFS |  |  |  |  |
| *Low risk* | 80 (77–84) | 87 (83–91) | 88 (84–92) |  |
| *Intermediate risk* | 70 (64–76) | 74 (69–80) | 86 (82–91) |  |
| *High risk* | 58 (50–67) | 60 (54–66) | 45 (40–52) |  |
| OS |  |  |  |  |
| *Low risk* | 88 (85–92) | 93 (90–96) | 91 (88–95) |  |
| *Intermediate risk* | 81 (76–86) | 84 (80–88) | 93 (90–96) |  |
| *High risk* | 69 (62–77) | 72 (67–78) | 65 (59–71) |  |

CI, confidence interval; COO, cell-of-origin; IPI, International Prognostic Index; OS, overall survival; PFS, progression-free survival.

**Supplemental Fig S1.** Schematic of analysis workflow.


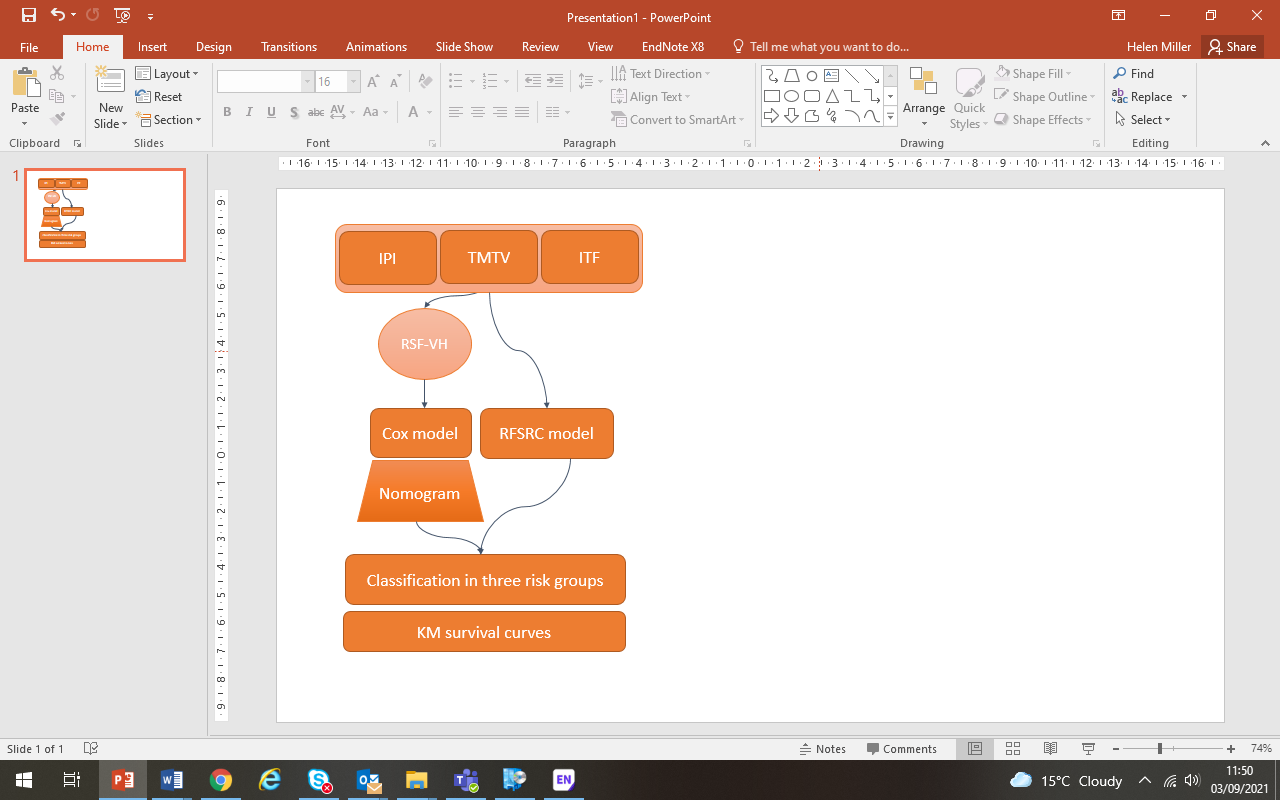


IPI, international prognostic index; ITF, image texture feature; KM, Kaplan-Meier; RF-SRC, random forests for survival, regression, and classification; RSF-VH, random survival forest-variable hunting; TMTV, total metabolic tumour volume.

**Supplemental Fig S2.** Nomogram obtained with Cox model for PFS of (A) the total population and (B) the COO subgroup.

| 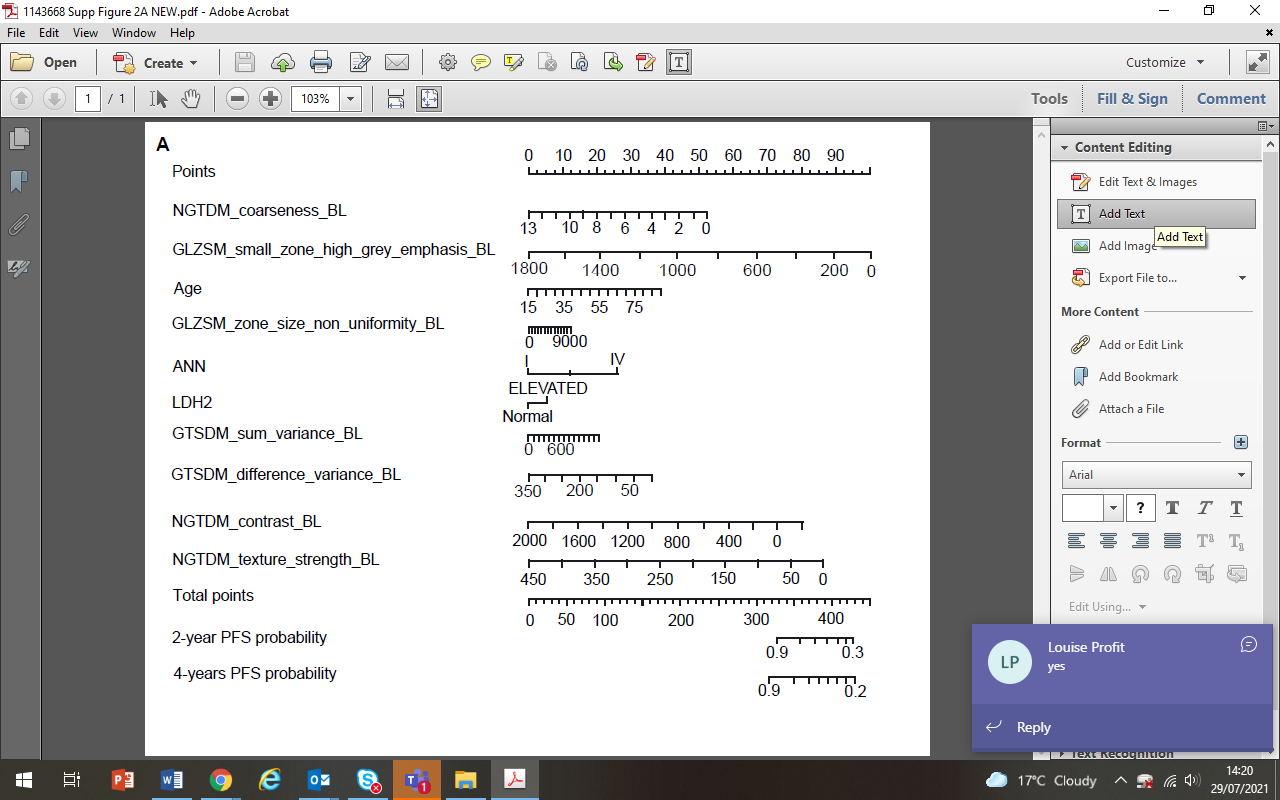 | 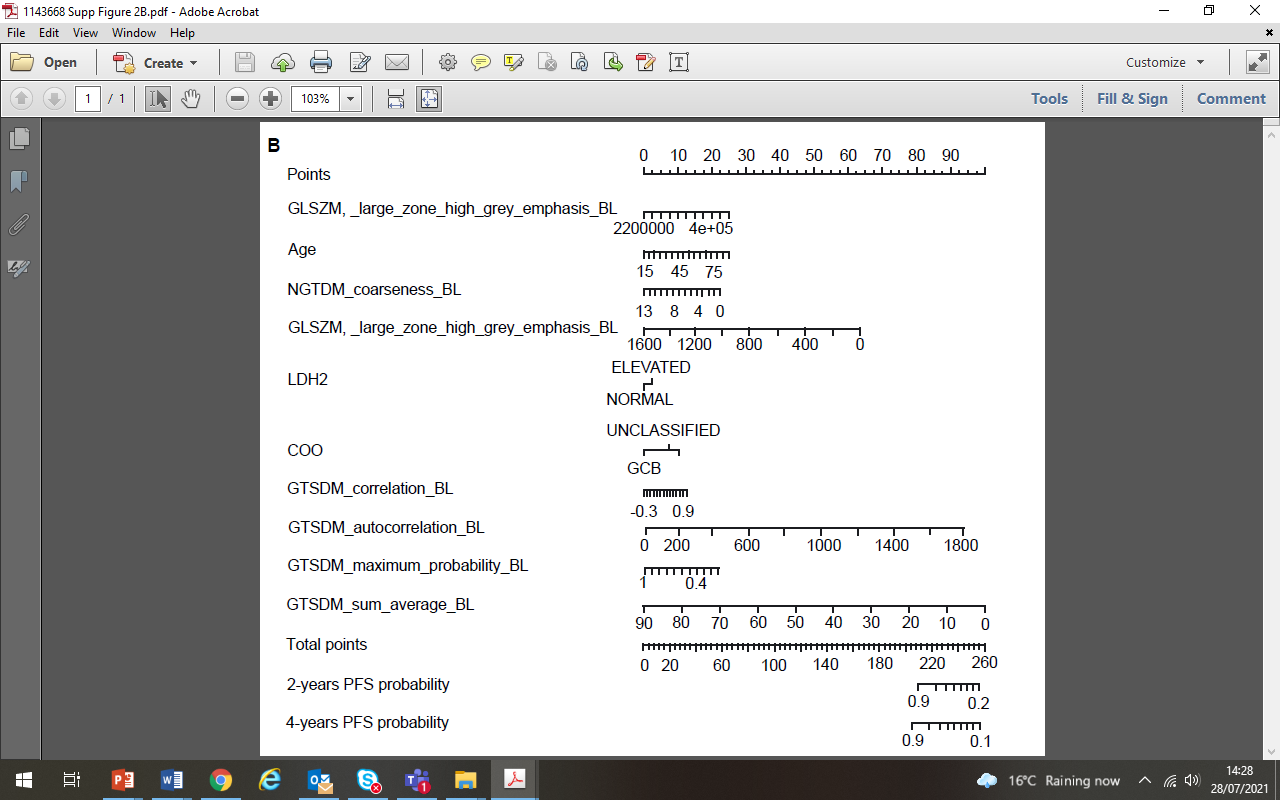 |
| --- | --- |

ANN, Ann Arbor stage; BL, baseline; COO, cell-of-origin; GCB, germinal centre B-cell; GLSZM, low grey-level zone length matrix; GTSDM, grey-tone spatial dependence matrices; LDH, lactose dehydrogenase; NGTDM, neighboring grey-tone difference matrix*;* PFS, progression-free survival; TMTV, total metabolic tumour volume.

**Supplemental Fig S3.** Kaplan-Meier PFS curves for the three risk groups as defined by (A) IPI, (B) Cox regression analysis and (C) random forest prediction model for all patients. IPI and random forest model are the same as in the main manuscript and are provided for comparison. **Note:** Dashed lines indicate the 95% confidence intervals.

| 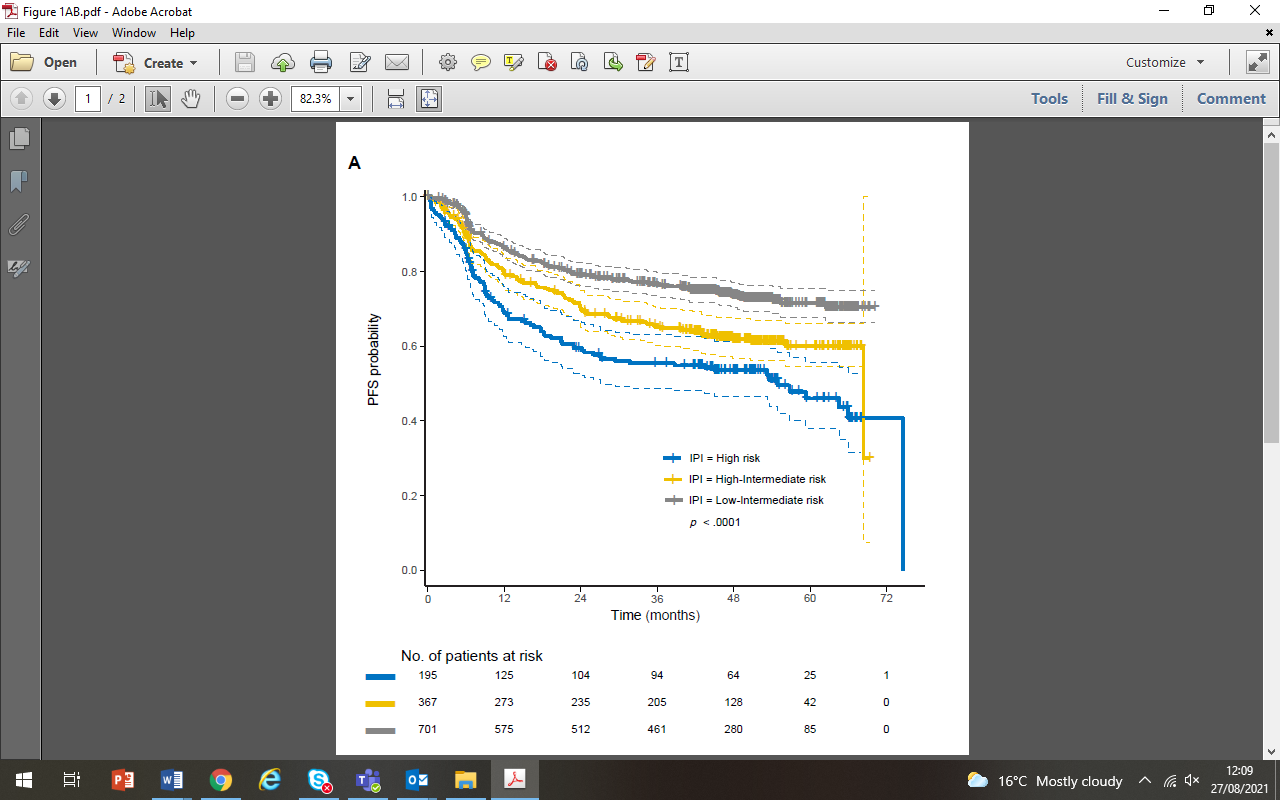 | 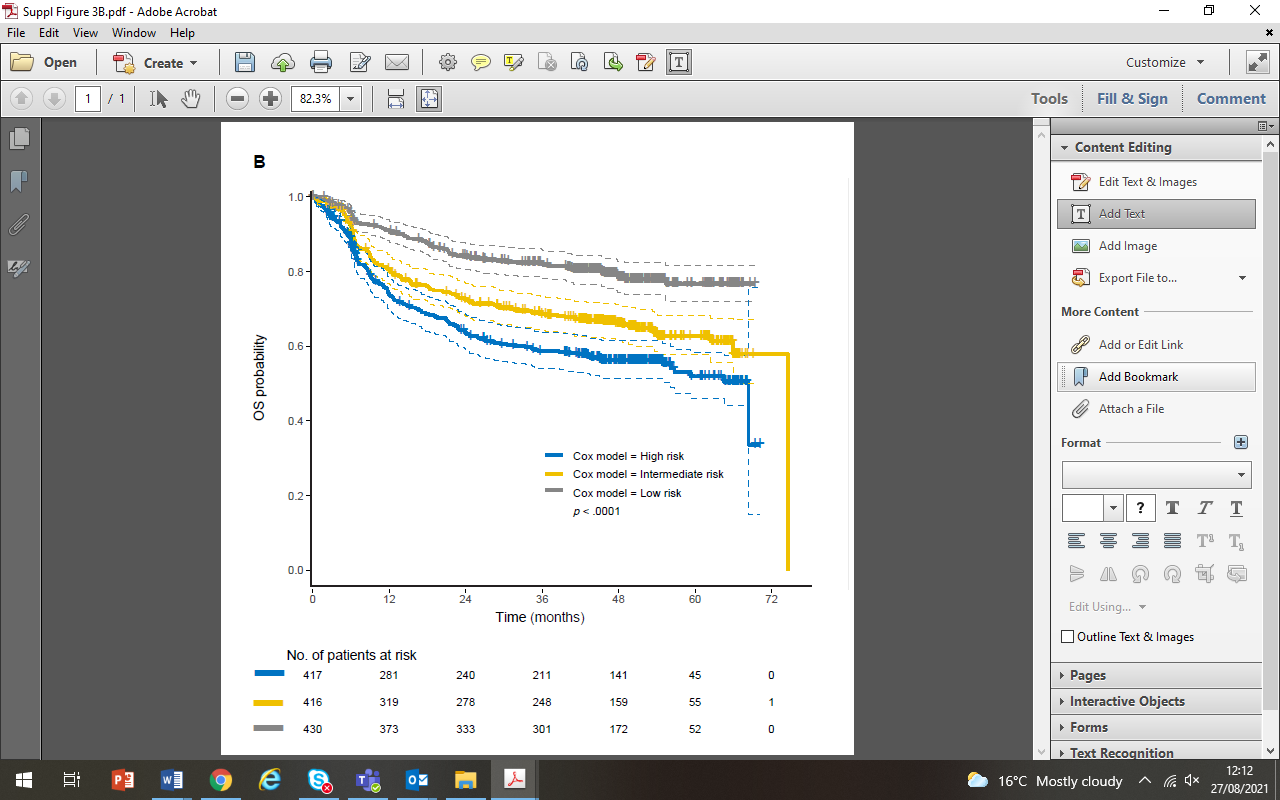 | 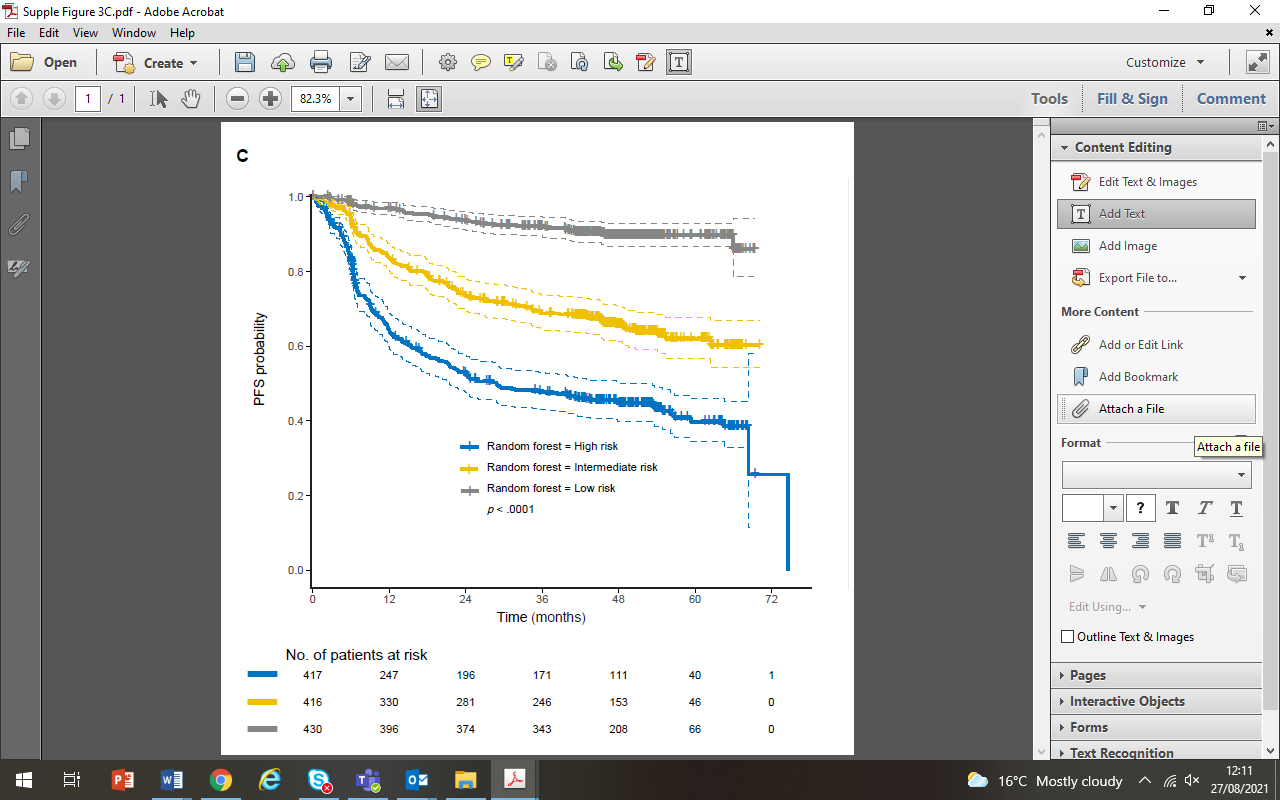 |
| --- | --- | --- |

| IPI, International Prognostic Index; PFS, progression-free survival. |  |  |
| --- | --- | --- |

**Supplemental Fig S4.** Kaplan-Meier OS curves for the three risk groups as defined by (A) IPI, (B) Cox regression analysis and (C) random forest prediction model for all patients. IPI and random forest model are the same as in the main manuscript and are provided for comparison. **Note:** Dashed lines indicate the 95% confidence intervals.

| 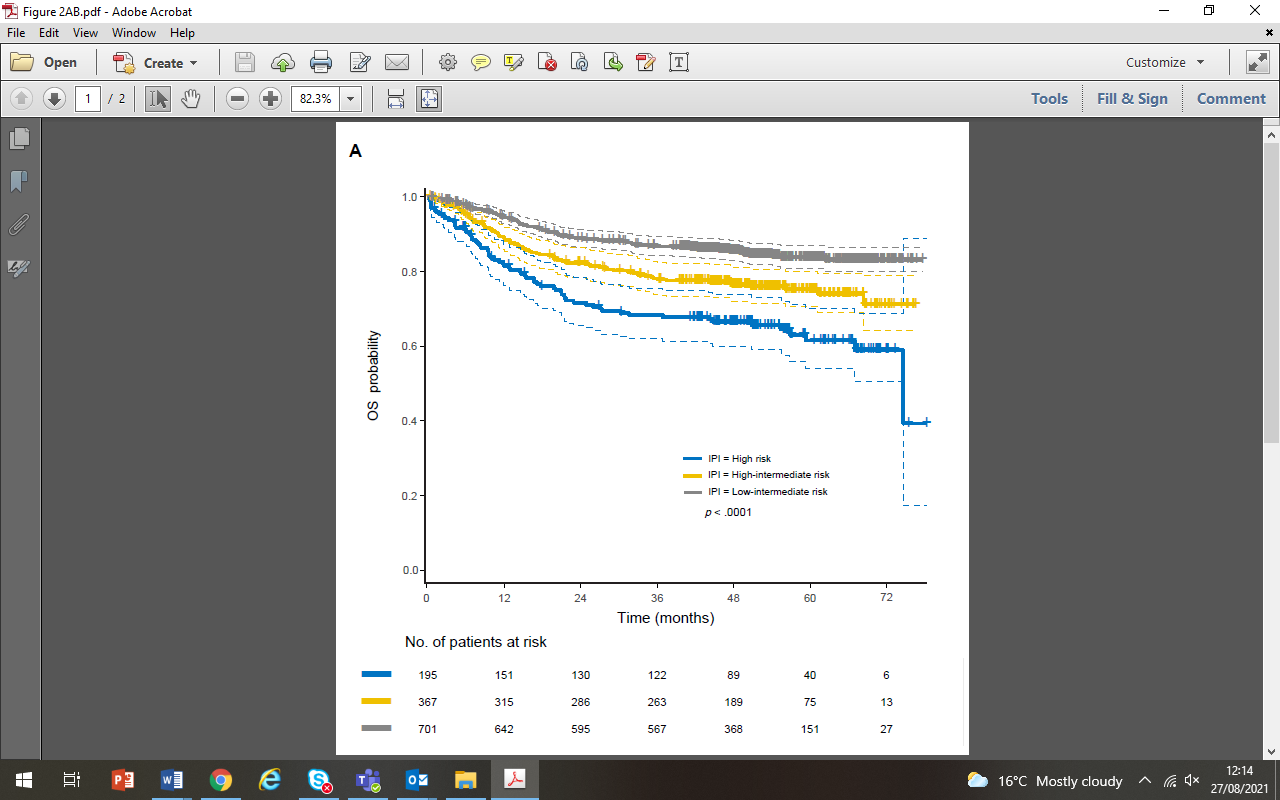 | 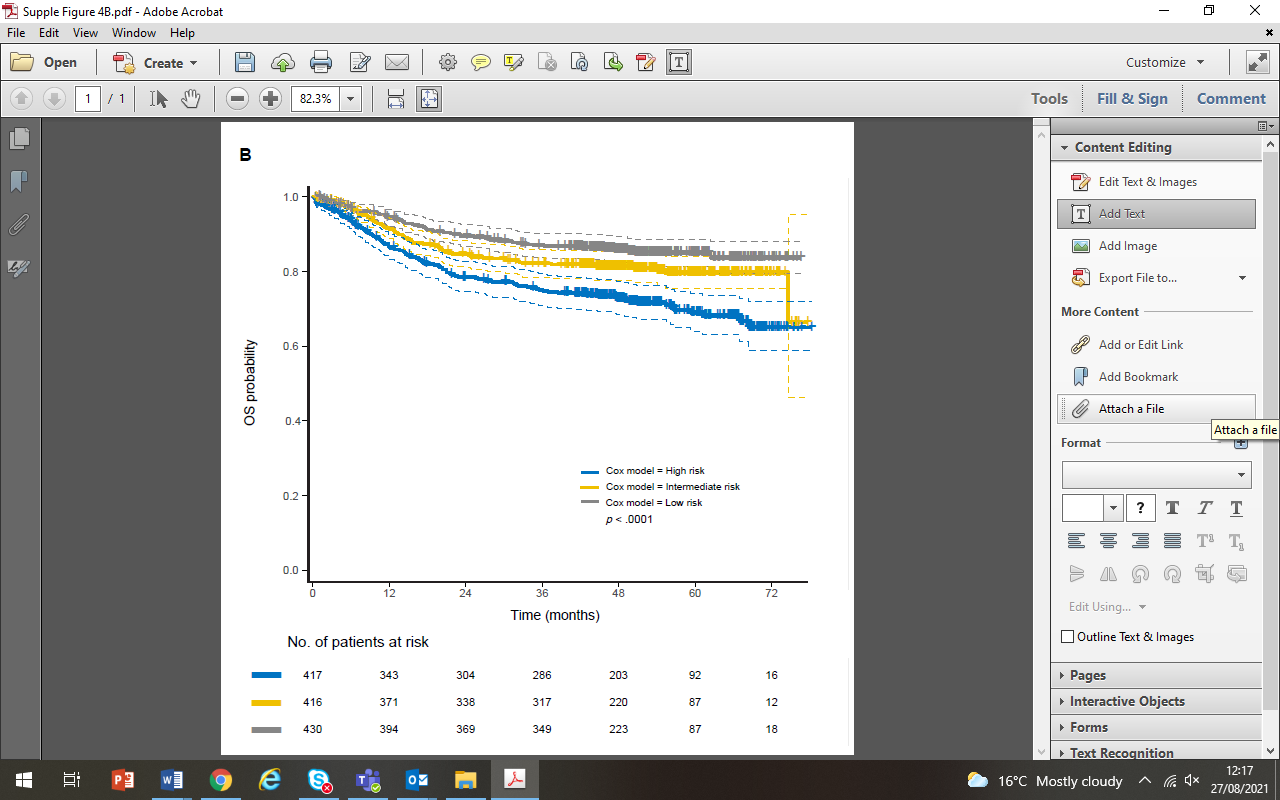 | 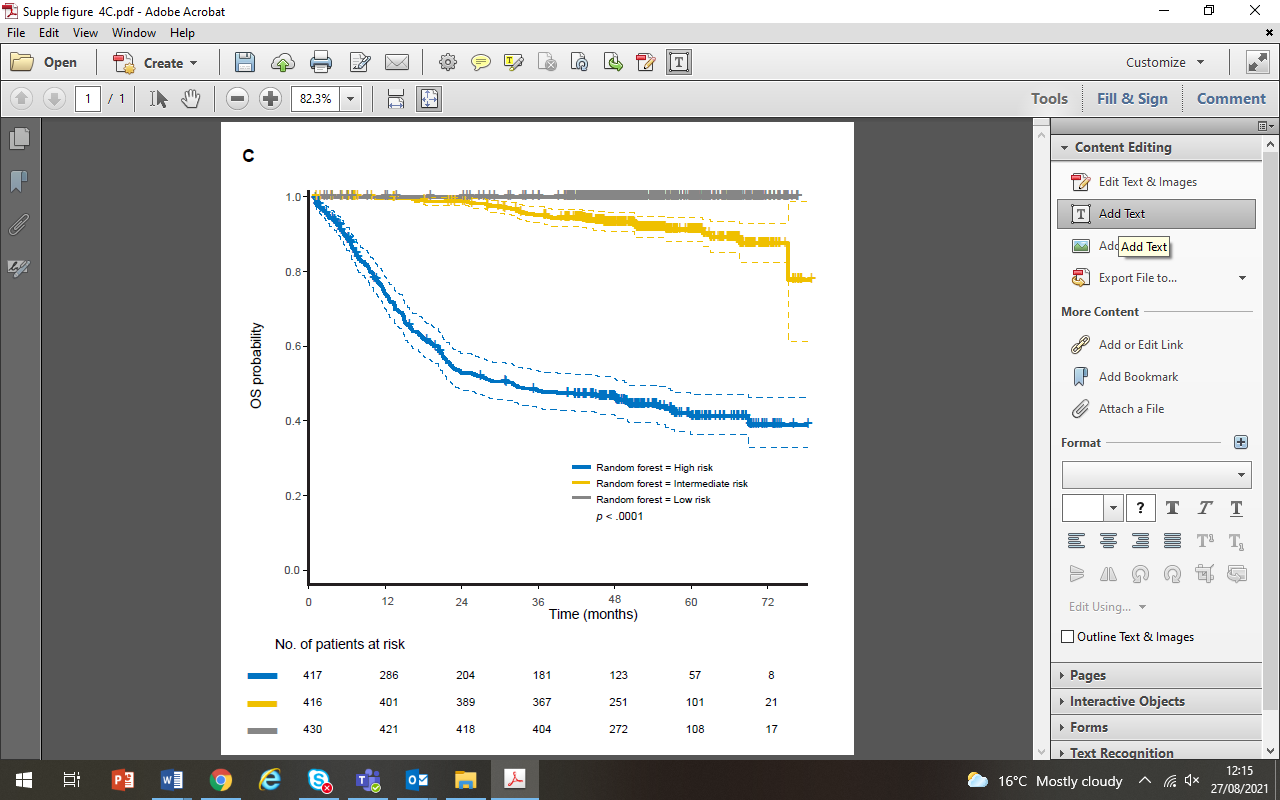 |
| --- | --- | --- |

IPI, International Prognostic Index; OS, overall survival.

**Supplemental Fig S5.** Kaplan-Meier PFS curves for the three risk groups as defined by (A) IPI, (B) Cox regression analysis and (C) random forest prediction model for the COO subgroup. IPI and random forest model are the same as in the main manuscript and are provided for comparison. **Note:** Dashed lines indicate the 95% confidence intervals.

| 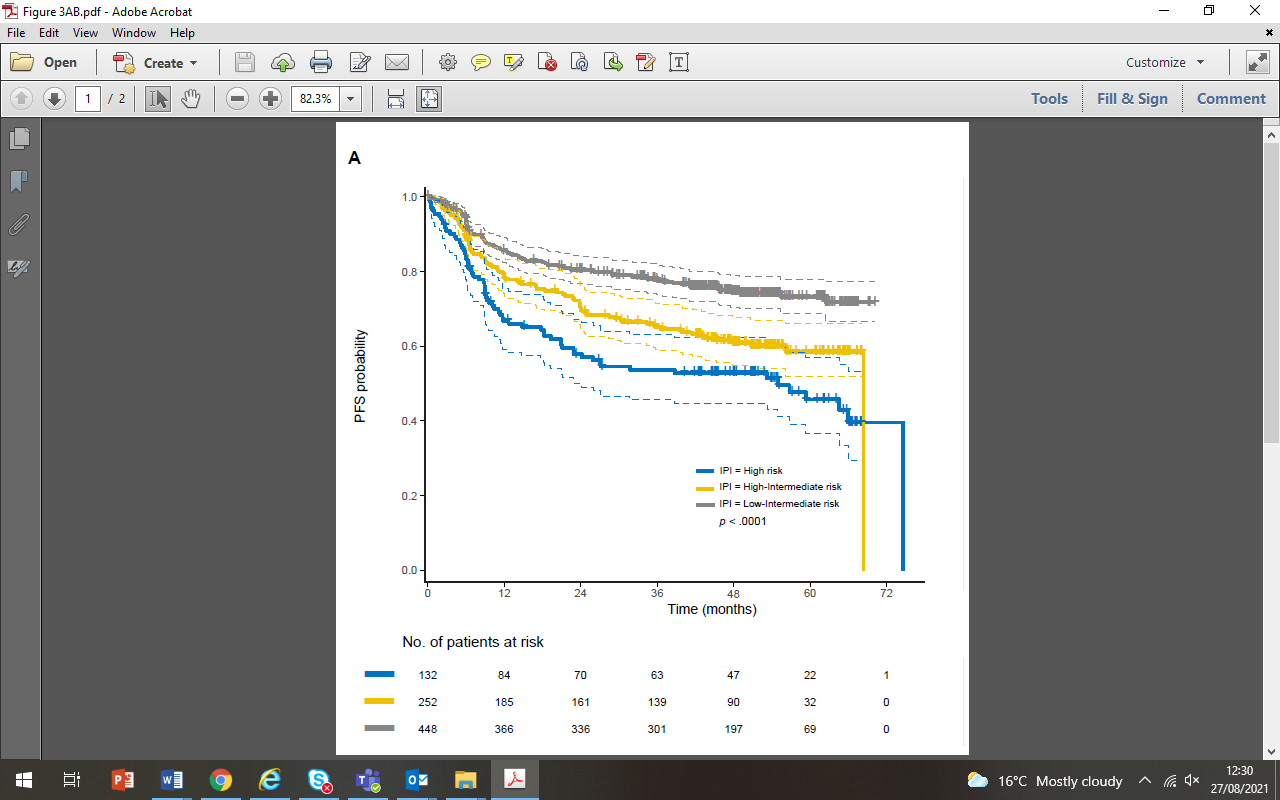 | 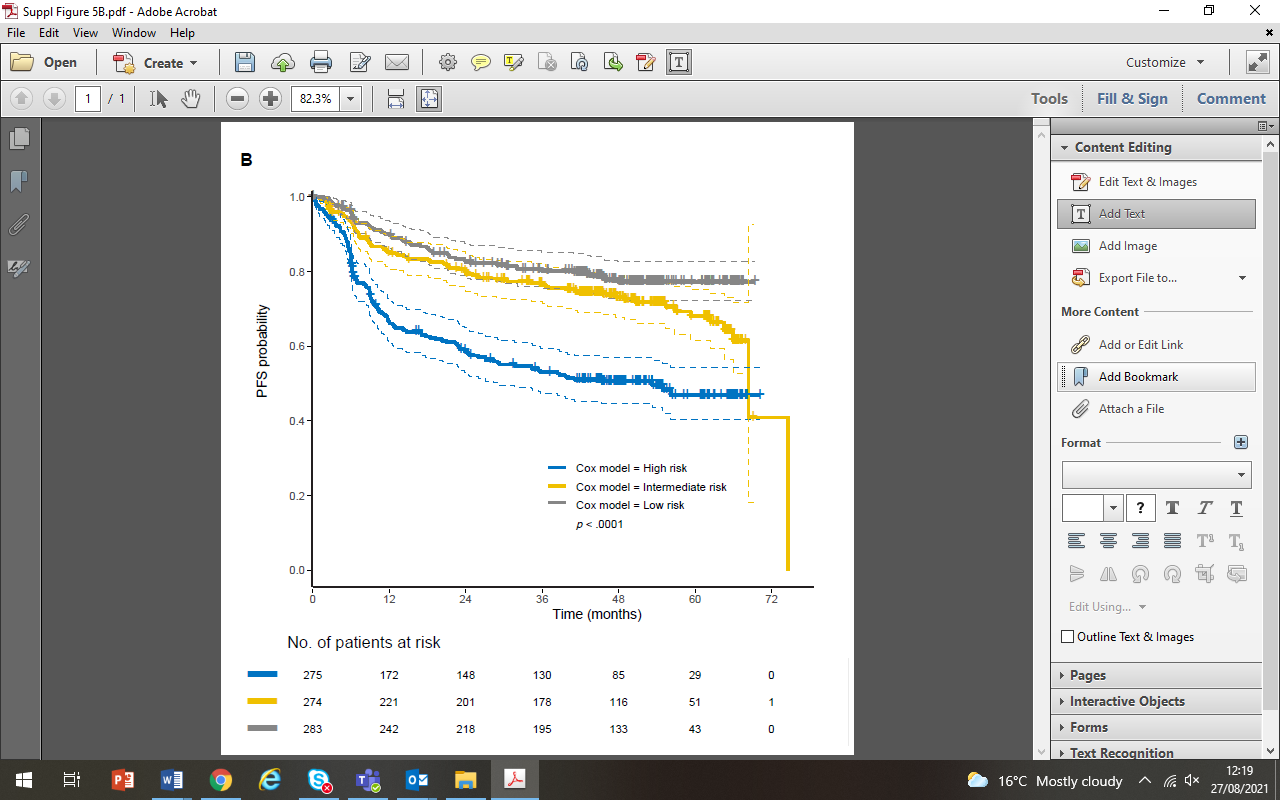 | 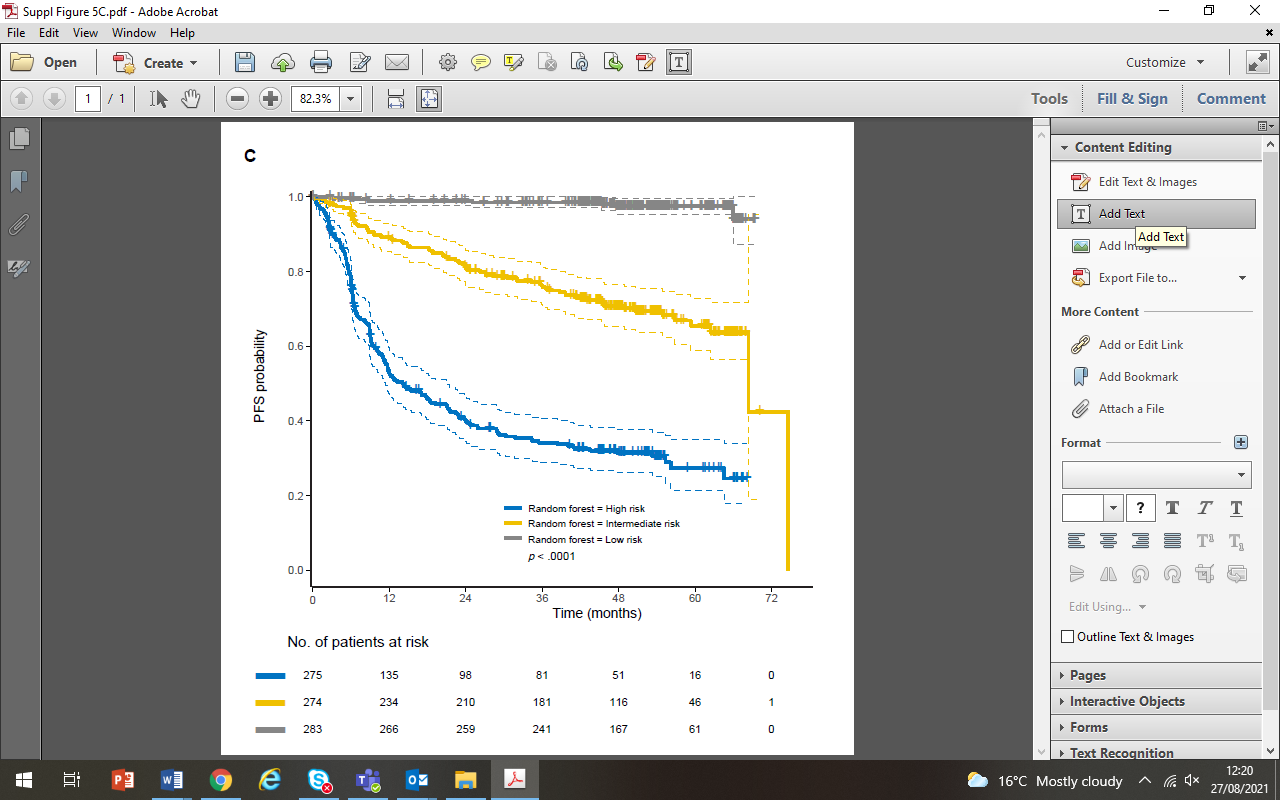 |
| --- | --- | --- |

COO, cell-of-origin; IPI, International Prognostic Index; PFS, progression-free survival.

**Supplemental Fig S6.** Kaplan-Meier OS curves for the three risk groups as defined by (A) IPI, (B) Cox regression analysis and (C) random forest prediction model for the COO subgroup. IPI and random forest model are the same as in the main manuscript and are provided for comparison. **Note:** Dashed lines indicate the 95% confidence intervals.

| 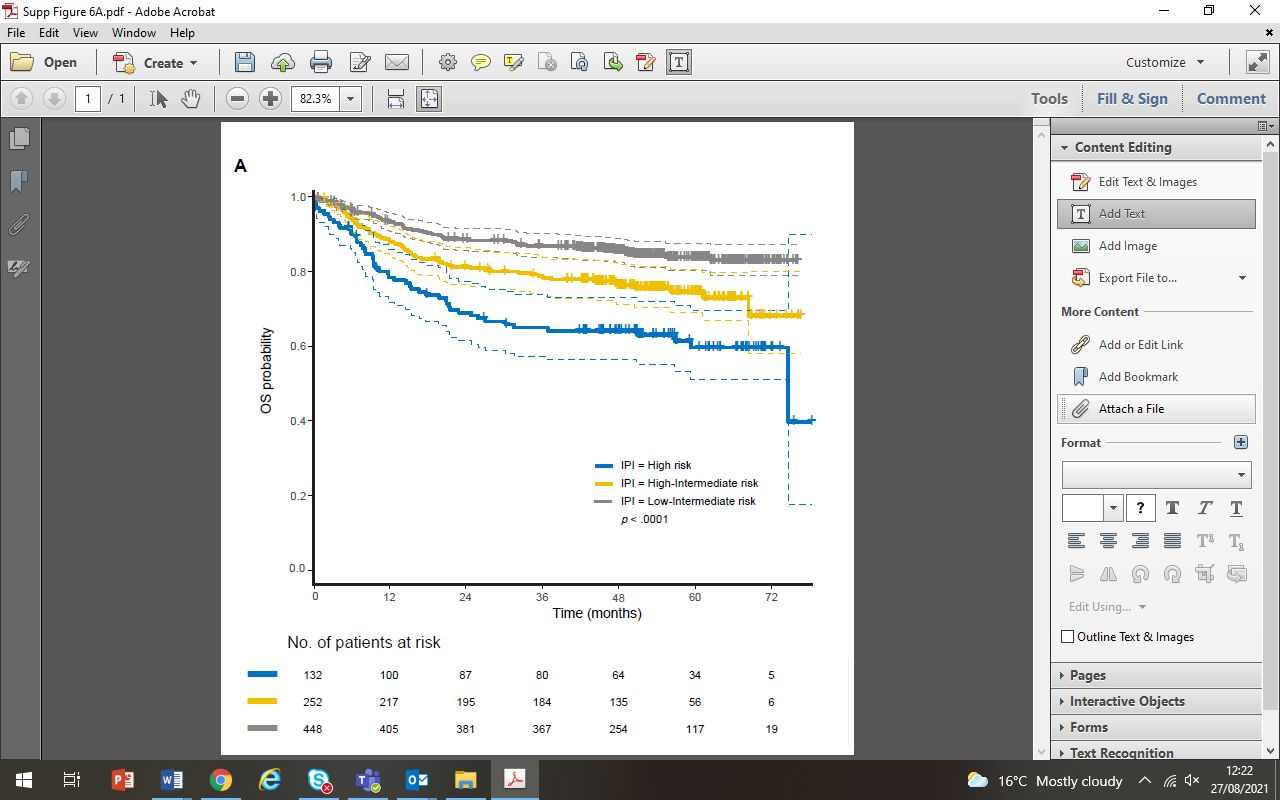 | 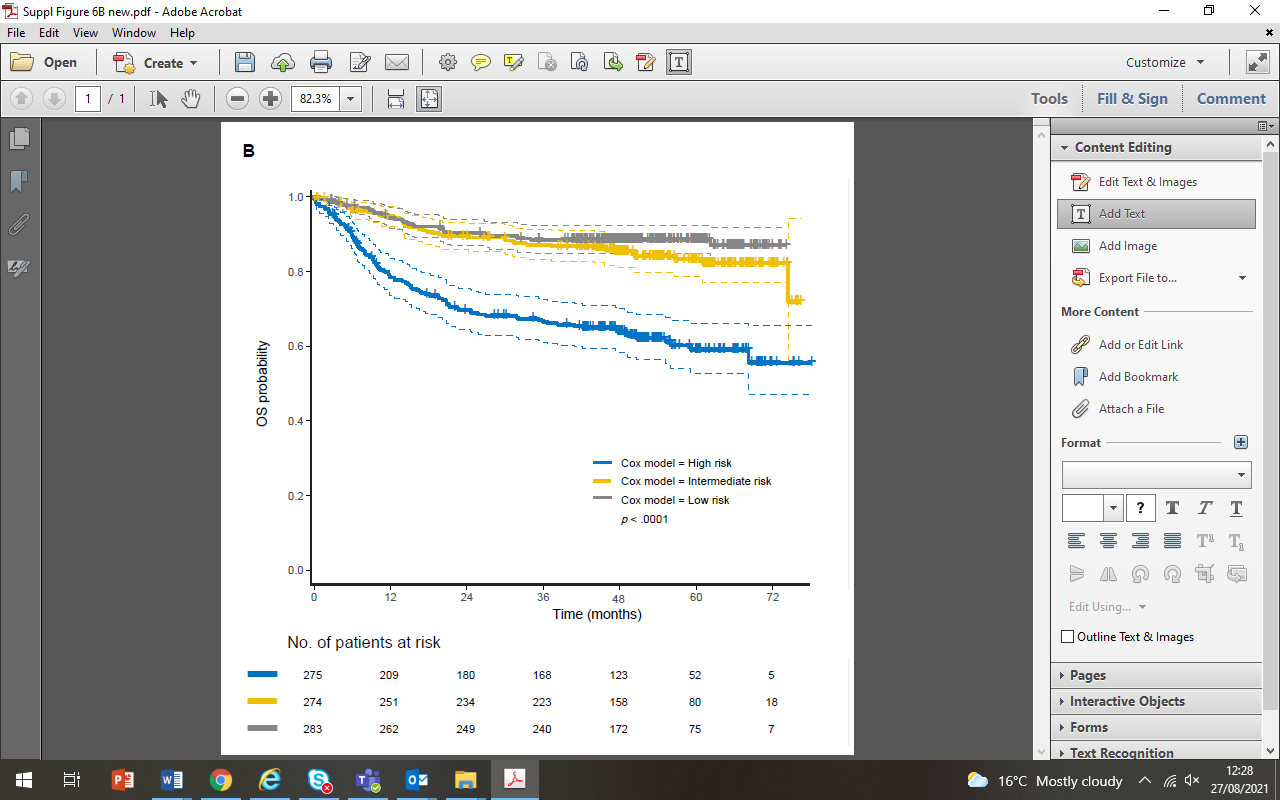 | 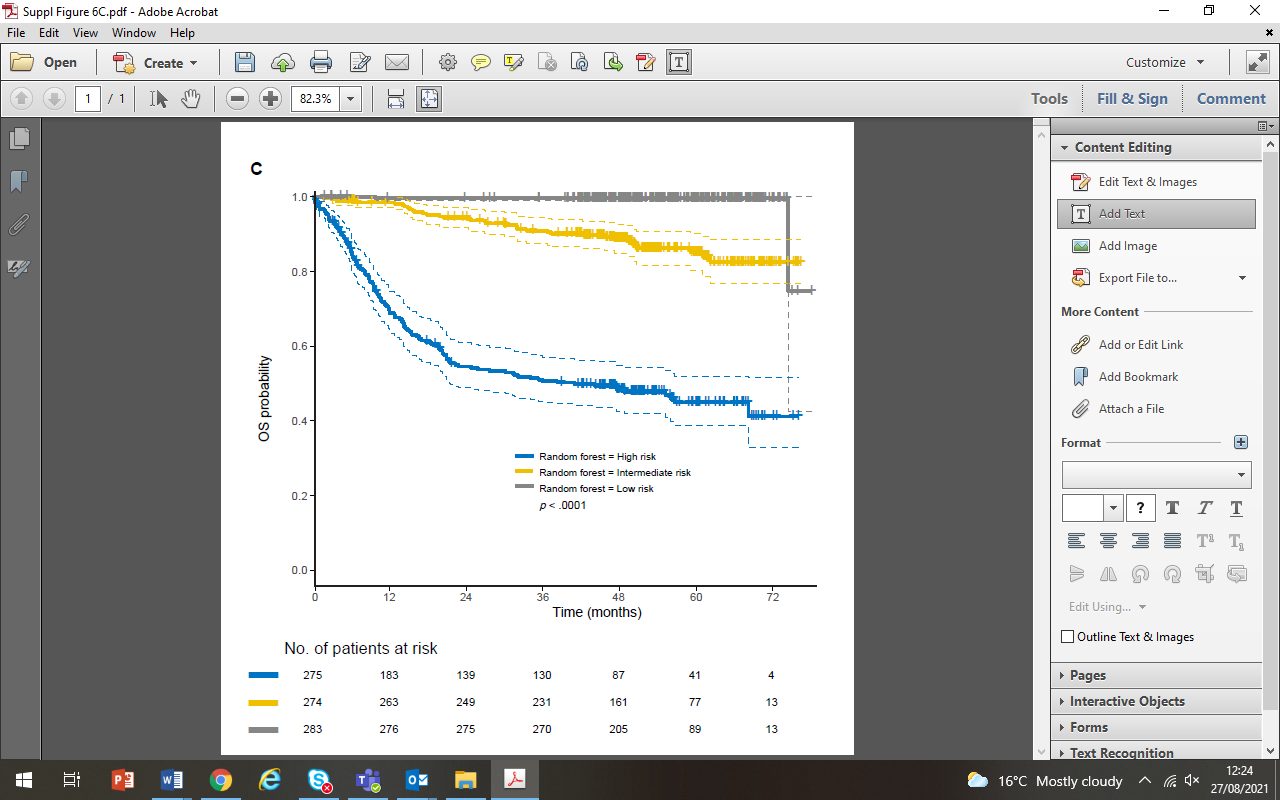 |
| --- | --- | --- |

COO, cell-of-origin; IPI, International Prognostic Index; OS, overall survival.
